# *Bagarius bagarius*, and *Eichhornia crassipes* are suitable bioindicators of heavy metal pollution, toxicity, and risk assessment

**DOI:** 10.1038/s41598-023-28313-9

**Published:** 2023-02-01

**Authors:** Mohammad Mahamood, Farmanur Rahman Khan, Farhana Zahir, Mehjbeen Javed, Saleh S. Alhewairini

**Affiliations:** 1 Department of Biology, Deanship of Educational Services, Qassim University, Buraydah, Saudi Arabia; 2 Department of Plant Production and Protection, College of Agriculture and Veterinary Medicine, Qassim University, Buraydah, Saudi Arabia; 3 Present Address: Department of Medical Elementology and Toxicology, Jamia Hamdard, New Delhi, 110092 India

**Keywords:** Ecology, Zoology, Ecology, Limnology

## Abstract

Water quality index (WQI) of Narora channel and health of endemic fish *Bagarius bagarius* and plant *Eichhornia crassipes*, district Bulandshahar, Uttar Pradesh, India were studied. Among the physicochemical properties of water, pH, D.O, Cr, Fe, Ni, and Cd were above the recommended standards. These factors lead to high WQI (4124.83), indicating poor quality and not suitable for drinking and domestic usage. In fish tissues, the highest metal load was reported in the liver (58.29) and the lowest in the kidney (33.73). Heavy metals also cause a lowering of condition indices. As expected, decreased serum protein (− 63.41%) and liver glycogen (− 79.10%) were recorded in the exposed fish. However, blood glucose (47.22%) and serum glycogen (74.69%) showed elevation. In the plant, roots (21.50) contained the highest, and leaves (16.87) had the lowest heavy metal load. Bioaccumulation factor (BAF) > 1, indicates hyperaccumulation of all metals. *E. crassipes* roots showed the highest translocation factor (TF) > 1 for Ni (1.57) and Zn (1.30). The high mobility factor (MF) reflected the suitability of *E. crassipes* for phytoextraction of Mn, Cd, Zn, Fe, Ni, and Cu. Moreover, *Bagarius* sp*.* consumption could not pose any non-cancer risk. Although, lower cancer risk can be expected from Ni and Cr.

## Introduction

The quality of water is of paramount importance for the survival and health of human beings as well as other flora and fauna. It is one of the determinants of life. However, it’s quality in developed and as well as in developing countries is greatly uneven, which may reflect their economic, social, and physiological status. Although, these countries do not have water shortage problems, but have poor water quality issues^1,2^. It is well known that the development of industries, urbanization, inappropriate agriculture practices, the ever-expanding population, etc., all have damaged the freshwater resources by discharging effluents, runoffs, and dumping wastes^3^. Islam^4^ determined trace metals in the deep and surface water of Korotoa river, Bangladesh. Rizwan et al.^5^ detected toxic metal concentrations from different environmental matrices. Emet Stream Basin is one of the important river systems of Turkey which was polluted by Harmancik Chromium Mines. The chromium (Cr) levels in the water of the basin and fish *Capoeta tinca*, *Squalius cii*, and *Barbus oligolepis* were found to be higher than the prescribed limits^6^. All such water quality issues can be curtailed only after periodic and systematic surveillance. The present investigation was undertaken to check the degraded water quality of the Narora channel at Narora (27° 30′ 0.00" N, 78° 25′ 48.00" E), district Bulandshahar, Uttar Pradesh, India. Surveillance of the Narora channel is quite important because it emerges from the middle Ganga reach which extends from the Narora to the district Ballia. This Narora channel supplies water almost to the entire village, crop fields of Narora and adjacent areas. Some investigations have found the poor water quality of the Ganga river at its different reaches^7–9^. However, to our knowledge, no investigations were made on this channel. In this region, the Ganga river and the Narora channel receive wastes from industrial and domestic sources, solid garbage is directly thrown at it, mass bathing, clothes washing, and defecation, etc. Heavy metals form the most common group of such persistent and non-biodegradable wastes^10,11^. The presence of heavy metals in water not only affects the quality of water but also makes it unfit for drinking and other use, economic loss, as well as limits the upgradation of living standards of the native community. Everywhere clean drinking water is in short supply that too in villages. Therefore, the local community depends on natural freshwater resources for all their needs. For that reason, it has become quite supreme to take organized steps to check quality of water. The water quality index (WQI) helps in the interpretation of water quality by associating complex data and giving rise to a score that report water quality status^2,12^. Moreover, living organisms and WQI can be utilized as bio-indicators to monitor the effect of pollution. Fishes are the chief organisms of any freshwater ecosystem therefore their health status shows the quality of water. It is also a fact that heavy metals cause stress. To overcome stress, fish utilize its energy reserves like carbohydrates, fats, and protein. Moreover, depletion or upregulation of energy reserves impacted the body indices, hepatosomatic index, etc. Recently Samim and Vasim^13^ highlighted changes in haematological variables like hemoglobin concentration, count of blood cells, oxygen-carrying capacity, etc. due to pollution. Tokatli^14^, find out high amounts of heavy metals in Gala Lake, Turkey with the help of diatoms and fish as bioindicators. Furthermore, the inhabiting flora also represents the bioaccumulation of heavy metals in different parts^15,16^. Translocation to shoots is restricted, however, heavy metal amounts can increase in roots and may go beyond 100,000 times more than the surrounding water^11,12^. Since aquatic plants bioaccumulate heavy metals hence they can be utilized to check the pollution in freshwater bodies^15,17^. *Eichhornia crassipes* is the most common freshwater tropical and subtropical plant. Almost more than a decades back, *E. crassipes* was cultured for ornamentation purposes, however, it spread at a fast rate if it found a suitable ambiance (https://www.cabi.org/isc/datasheet/20544#tosummaryOfInvasiveness). It can withstand extreme conditions of temperature, pH, toxic substances, etc.

In the present investigation, the area under study is rural, where the residing community or the natives depend entirely on natural freshwater resources for drinking, and other purposes. Therefore, the target is to discuss the suitability of Narora channel water for consumption by locals and for other domestic uses based on WQI physicochemical variables. Moreover, the accumulation in endemic fish *Bagarius bagarius* and *E. crassipes* plant were also utilized as bio-indicators of the Narora channel. Fish *Bagarius sp.* is a carnivore fish and so it can feed on zooplankton, molluscs, insects, and even piscivore. Besides, it also shows antisocial behavior in captivity so also known by the name of devil fish. It prefers to live in fast flowing waters. Furthermore, fish condition indices, metal pollution index (MPI), and harmful impact of heavy metals on energy sources such as glucose, glycogen, and protein amounts were measured. In addition to this the bioaccumulation factor (BAF), translocation factor (TF), and mobility factor (MF) were also measured in *E. crassipes*. Since the residents of the investigated area and also professional fishermen catch fish from this channel hence, human health risk assessment was also evaluated.

## Results and discussion

### Analytical method validation

The results of the precision study with relative standard deviation (RSD), and accuracy are shown in Table 1. Through the precision study we found the value of RSD as less than 5%. Moreover, accuracy was done with percent recovery experiments. The results showed that the percentage recoveries for spiked samples were in the range of 95.7–103.7%.

**Table 1:** Shows percent (%) recovery and relative standard deviation.

Metal ions	Amount spiked (µg g^−1^)	Amount found (µg g^−1^)	% Recovery	RSD
Cr	0	0.45	–	2.29
	5	5.29	96.6	3.92
	10	10.48	100.2	4.71
Mn	0	0.15	–	3.88
	5	5.04	97.6	4.85
	10	10.17	100.1	1.56
Fe	0	1.44	–	4.93
	5	6.62	103.7	1.84
	10	11.01	95.7	4.60
Ni	0	1.70	–	1.42
	5	6.67	99.1	3.48
	10	11.49	97.8	3.72
Cu	0	0.26	–	4.62
	5	5.43	103.6	4.46
	10	10.49	102.4	4.07
Zn	0	0.77	–	3.14
	5	5.72	98.9	0.36
	10	10.56	97.9	1.10
Cd	0	0.92	–	1.39
	5	6.02	101.8	1.69
	10	10.95	100.3	0.24

Blank cells indicate real sample.

### Physicochemical properties and water quality index

The investigations of the water quality properties of the Narora channel are shown in Table 2. The temperature, TDS, turbidity, and alkalinity were within the standards of the country^18^ and WHO^19^ (taken from UNEPGEMS). While pH and dissolved oxygen (D.O) were above the recommended standards indicating poor water quality. Moreover, the detected heavy metals were in the following order Ni > Fe > Cd > Zn > Cr > Cu > Mn. Among these heavy metals Mn, Cu, and Zn were within the recommended limits whereas Cr, Fe, Ni, and Cd were crossing the limits^18^ contributing to the poor quality. Furthermore, the WQI calculation will give more insights into the overall quality of water as it explains the combined effect of several physicochemical properties^12^. Its calculation is done simply by converting numerous variables of water quality into a single number^12,20^. In addition to this, WQI simplifies all the data and helps in clarifying water quality issues by combining the complex data and producing a score that shows the status of water quality^2,12,21^. The WQI classifies water quality status into five groups such as if WQI < 50 indicates excellent quality; WQI = 50–100 designates good quality; WQI = 100–200, shows poor quality; WQI = 200–300 reflects inferior quality; and if WQI is above 300 then it is unfit for drinking^12^. In the current study, the WQI was calculated to be 4124.83, which did not fall in the set WQI groups. The WQI results show that Narora channel water in the investigated rural area is unsuitable for drinking as well as other domestic purposes. This too high WQI value at Narora channel water could be correlated to Ni and Cd which proves to be the main culprits due to their high qi values of 885.04 and 3196.26, respectively which leads to high SI and consequently high WQI. The high Ni and Cd content may be due to the effluents of several types of sources like sugarcane and iron factories, cement dust, mechanical workshops, and agricultural activities, etc. near the bank which drain their partial or untreated effluents into the channel. The wastes from domestic sources further contribute to pollution. In the upper Ganga region from Brijghat to Narora very poor water quality was reported by Prasad et al.^7^ Tabrez et al.^2^ also found very high WQI in Kshipra River at Dewas segment, Madhya Pradesh, India. Giao et al.^22^ reported worse quality of water in low-lying areas of the Vietnamese Mekong Delta. The other rural, as well as urban regions of India, also do not meet the national guidelines of water quality in natural freshwater resources^3,12,23^. Furthermore, the rural regions of the Colombian Caribbean represent poor water quality^24^. Such investigations highlighting water quality problems become more meaningful when integrated with the assessment of adverse impacts on the health outcomes of bio-indicator organisms inhabiting the ambiance. Therefore, endemic fish *Bagarius sp.* and a plant *E. crassipes* were chosen for further investigations.

**Table 2:** Water quality properties of Narora channel, Bulandshahar, Uttar Pradesh, India.

Parameters*	^a^Narora channel water values	^b^Indian standards/WHO	wi	Wi	qi	SI
Temperature	20 ± 0.001	20–30	4	0.083	66.66	5.53
pH	5 ± 0.001	6.5–8.5	4	0.083	58.82	4.88
D.O	5.53 ± 0.01	6–8	5	0.104	69.12	7.18
Alkalinity (CaCO_3_)	415 ± 0.39	200–600	2	0.041	69.16	2.83
Turbidity	5.2 ± 0.01	10	2	0.041	52	2.13
TDS	610 ± 2.5	500–2000	4	0.083	30.5	2.53
Cr	0.454 ± 0.001	0.05	5	0.104	9.08	0.94
Mn	0.156 ± 0.001	0.1–0.3	4	0.083	52	4.31
Fe	1.447 ± 0.01	0.3–1.0	4	0.083	144.7	12.01
Ni	1.702 ± 0.01	0.02	5	0.104	8510	885.04
Cu	0.265 ± 0.001	1.5	3	0.062	17.66	1.09
Zn	0.778 ± 0.001	5–15	1	0.020	5.18	0.10
Cd	0.922 ± 0.001	0.003	5	0.104	30,733.33	3196.26
			Σwi = 48			WQI = 4124.83

^a^All parameters are presented as mean ± SEM (n = 3).

^b^Range of limits where value limit shows desirable standard.

*Temperature was measured in °C and turbidity in NTU whereas D.O, alkalinity, TDS, and all metals in mg/L.

### Bioaccumulation and MPI in *Bagarius bagarius*

The average body length from the snout to the tip of the caudal fin of the exposed fish was found to be 22.7 ± 0.9 cm, the average weight was 145.73 ± 1.3 g and that of the reference fish was 18 ± 0.6 cm and 128 ± 0.96 g respectively.

**Figure 1:**
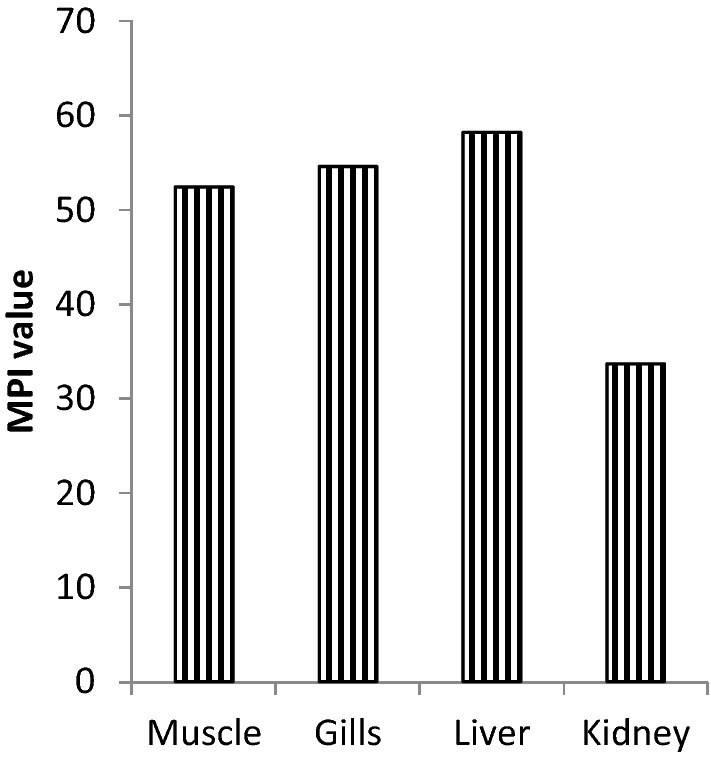
MPI values in *Bagarius bagarius*.

Fish *Bagarius sp.* accumulated significant concentrations of heavy metals in the muscle, gills, liver, and kidney (Table 3). In muscle, Cd (94 mg/kg.dw) showed the highest accumulation, and Mn (12.9 mg/kg.dw) accumulated the lowest. Likewise, in gills (96.3 mg/kg.dw) and kidney (72 mg/kg.dw), Cd accumulation was highest while Mn shows lowest accumulation 13.45 mg/kg.dw and 9 mg/kg.dw in both the organs respectively. In the liver, Cu (102 mg/kg.dw) accumulation was highest and Mn (20 mg/kg.dw) lowest. However, the MPI calculation showed that the liver (58.29) has the highest burden of heavy metals among all the tissues followed by the gills (54.66), muscle (52.50) and the lowest load in the kidney (33.73) (Fig. 1). The interpretation derived from the bioaccumulation and MPI results is that liver is the most vulnerable target organ of heavy metals may be because of its involvement in all the metabolic processes. It also takes part in the detoxification of toxicants^25^. Moreover, gills and muscles proved to be the next target organs for heavy metal toxicity. Seemingly, the liver and gills were unable to excrete these heavy metals fully because they might have bound with the macromolecules and enzymes. Muscle contained high concentrations of metals which may be due to the reason that their metabolization occurs in the liver and part of them binds with the myoglobin and remain in the muscle tissue. The lowest heavy metal load in the kidney indicates that the kidneys function efficiently to remove these metals. Similar results were also observed by Khan et al.^23^ and Mahamood et al.^3^ in *Oreochromis niloticus* and *Labeo rohita* living in the river Yamuna repectively. Moreover, Tabrez et al.^11^ also found liver and gills as target organs in the same genus *Mystus tengara* and *vittatus*. Kose et al.^26^ reported higher metal levels in the gills and liver of fish *Carassius gibelio* collected from dam lakes and Sakarya river, Turkey.

**Table 3:** Heavy metal concentrations in *Bagarius bagarius* tissues (mg/kg.dw).

Organs	Cr	Mn	Fe	Ni	Cu	Zn	Cd
Muscle	_a_49.95 ± 0.001^d^	_b_12.9 ± 0.01^e^	_c_41.45 ± 1.0^a^	_b_68.2 ± 1.1^d^	_b_83.8 ± 1.6^b^	_bc_76.7 ± 1.7^bc^	_ab_94 ± 1.8^a^
Gills	_c_31.2 ± 0.12^f^	_bc_13.45 ± 0.1^g^	_a_69.7 ± 1.2^e^	_a_75.15 ± 2.3^cd^	_c_75.35 ± 1.3^c^	_a_91.5 ± 1.96^ab^	_a_96.3 ± 2.2^a^
Liver	_b_38 ± 0.01^f^	_a_20 ± 0.31^g^	_ab_65 ± 1.8^d^	_c_59 ± 1.1^e^	_a_102 ± 2.2^a^	_b_81 ± 1.3^c^	_a_95 ± 1.7^b^
Kidney	_d_16 ± 0.01^f^	_d_9 ± 0.01^g^	_d_35 ± 0.89^e^	_d_51 ± 1.0^c^	_d_56 ± 0.9^b^	_d_48 ± 0.01^d^	_c_72 ± 1.0^a^
Allowable concentrations	0.491 USEPA (2000)	1.0 (WHO/FAO 1989)	100 (WHO/FAO 1989)	70–80 (USFDA 1993b)	30 (WHO/FAO 1989)	40/100 (WHO/FAO 1989)	0.05–3 (European Union 2006)

All values are presented as mean ± SEM (n = 15).

Superscripts and subscripts represents significant differences along the row and column respectively.

Significance was tested at p < 0.05.

#### Condition indices

Various condition indices of fish *Bagarius sp.* are given in Table 4. In the present study lower values of condition factor (K), hepatosomatic index (HSI), and kidney somatic index (KSI) were found as compared to the reference fish. These condition indices present a simple tool to surveil the health of fish in field studies. The most common among them are K, HSI, and KSI. K represents the general well-being of the fish and the low value of K shows inferior environmental quality. Moreover, HSI relates the weight of the liver to the body weight of fish. It gives more precise information relating to the function of the liver in response to the environment. Furthermore, kidneys play excretory, endocrine, hematopoietic as well as reticuloendothelial roles. Therefore, KSI also helps in determining the health of fish.

**Table 4:** Condition indices of fish *Bagarius bagarius*.

Condition indices	Exposed fish	Reference fish
K (g/cm^3^)	1.24	2.19
HSI	1.71	2.73
KSI	1.92	2.34

### Glucose, glycogen, and protein assays

**Figure 2:**
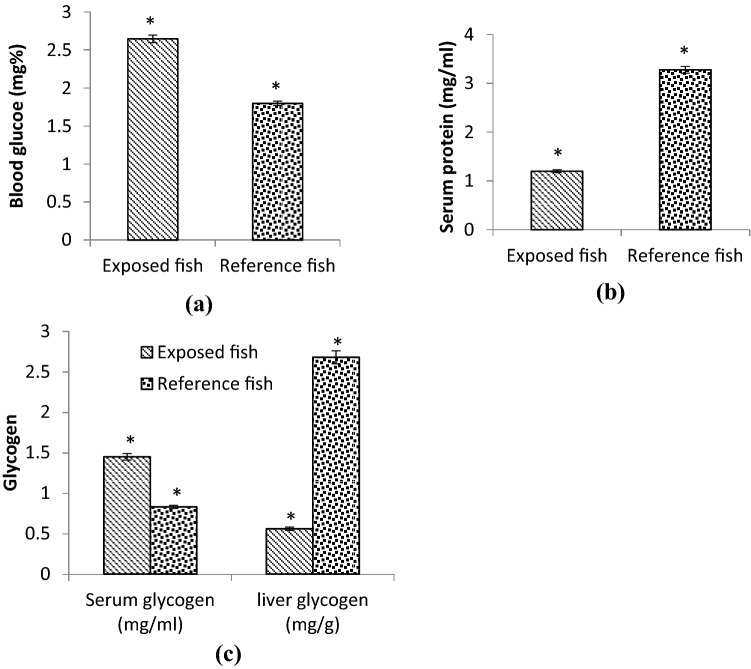
Illustrates the (**a**) blood glucose, (**b**) serum protein, (**c**) glycogen levels in serum and liver of exposed and reference *Bagarius sp.* Significance was checked at p < 0.05.

Blood based biomarkers are very informative in predicting the health of the fish or the entire population therefore, they are routinely used in biomonitoring studies^27^. In the present investigation, an increase (47.22%) was observed in the glucose levels in blood and serum glycogen (74.69%) in exposed *Bagarius sp.* However, the serum protein and liver glycogen concentrations got lowered by − 63.41% and − 79.10% in the exposed fish than in the reference fish (Fig. 2). It is well-known that heavy metals generate reactive oxygen species which cause stress by influencing several physiological processes. Carbohydrates and protein are energy sources. Glucose provides instant energy whereas glycogen is the reserve energy. So during stress conditions increase in glucose and serum glycogen indicates their utilization and mobilization from other tissues to the blood. Moreover, the decrease in serum protein and liver glycogen is also pointing in this direction. It is also reported that when glucose is in short supply in the body, a non-carbohydrate source would metabolize to glucose which could lead to its higher levels. Recently, Tabrez et al.^2^ reported depletions of all energy sources, glucose, glycogen, and protein in the serum of *Labeo rohita* living in the polluted Kshipra River. Bhilave et al.^28^ also found lower levels of glucose, glycogen, and protein under the effect of chronic heavy metals exposure. Lately, in *Heteropneustes fossilis* the As_2_O_3_ and PbCl_2_ exposure lead to disturbance in the carbohydrate metabolism^29^.

### Heavy metals uptake by *Eichhornia crassipes*, bioaccumulation factor, transfer factor and mobility factor

**Figure 3:**
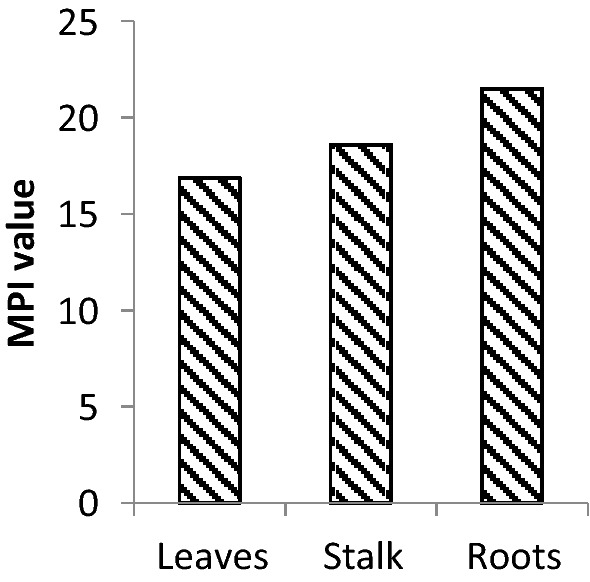
MPI values in *E. crassipes*.

Like inhabiting fauna, the flora also bioaccumulates heavy metals in different parts. The bioaccumulation data of *E. crassipes* leaves, stalk, and roots are presented in Table 5. This plant grows rapidly in polluted waters. The leaves, stalk, and roots accumulated the highest amounts of Cd 56 mg/kg.dw, 75 mg/kg.dw, and 81 mg/kg.dw respectively, while the Cr showed the lowest accumulation in all these parts 1 mg/kg.dw, 1.2 mg/kg.dw, and 1.8 mg/kg.dw respectively. According to MPI calculation (Fig. 3), the roots (21.50) contained the highest heavy metal load followed by the stalk (18.60) and then leaves (16.87). The high metal burden in roots pointed towards their habitat that they always remained immersed directly in the surrounding water. The plant part which is farther from the medium contained a lower load. Recently, Tabrez et al.^2^ and Singh et al.^15^ also found similar results in *E. crassipes*. The BAF, TF, and MF of *E. crassipes* are presented in Table 6. The highest BAF was reported for Mn and the lowest for Cr. The highest TF was found for Ni (1.57), and the lowest for Cu (0.66), Zn (1.30) also had TF above 1, whereas the rest of the heavy metals had comparable TF and it was below 1. Furthermore, the maximum MF values were observed for Mn for both roots to stalk (324.35) as well as stalk to leaves (211.53). However, it followed the order as Mn > Cd > Cu > Zn > Fe > Zn > Ni > Cr from root to stalk; and Mn > Cd > Zn > Cu > Fe > Ni > Cr from stalk to leaves.

**Table 5:** Heavy metal concentrations in *Eichhornia crassipes* (mg/kg.dw).

Plant parts	Cr	Mn	Fe	Ni	Cu	Zn	Cd
Leaves	_b_1 ± 0.001^g^	_c_35 ± 1.1^b^	_c_28 ± 0.9^c^	_c_3.9 ± 0.001^f^	_c_7.9 ± 0.01^e^	_b_23 ± 0.5^d^	_c_56 ± 1.4^a^
Stalk	_b_1.2 ± 0.001^g^	_b_33 ± 1.0^c^	_b_32 ± 1.2^cd^	_a_15 ± 1.0^e^	_b_13.2 ± 0.8^f^	_a_41 ± 0.66^b^	_b_75 ± 1.3^a^
Root	_a_1.8 ± 0.001^g^	_a_50.6 ± 1.8^b^	_a_43 ± 1.0^c^	_ab_14.5 ± 1.0^f^	_a_21 ± 0.21^de^	_b_22 ± 0.02^d^	_a_81 ± 1.7^a^
Allowable concentrations	0.491 USEPA^30^	1.0 WHO/FAO^31^	100 WHO/FAO^31^	70–80 USFDA^32^	30 WHO/FAO^31^	40/100 WHO/FAO^31^	0.05–3 European Union^33^

All values are presented as mean ± SEM (n = 8).

Superscripts indicates significant differences along the row and subscripts along the column.

Significance was tested at p < 0.05.

**Table 6:** Bioaccumulation factor (BAF), transfer factor (TF), and mobility factor (MF) in plant *E. crassipes*.

Heavy metal	BAF	TF	MF	
			Root-stalk	Stalk-leaves
Cr	2.92	0.74	3.96	2.64
Mn	253.39	0.78	324.35	211.53
Fe	23.72	0.79	29.71	22.11
Ni	13.41	1.57	8.51	8.81
Cu	52.94	0.66	79.24	49.81
Zn	36.83	1.30	28.27	52.69
Cd	76.63	0.87	87.85	81.34

These factors BAF, TF, and MF are utilized to monitor the level of anthropogenic pollution in plants and their surrounding medium^2,15,32,34,35^. BAF shows the concentrations of heavy metals bioaccumulated by plants from the water. If the BAF > 1 it indicates hyperaccumulation^36^. So, in the present study, all the concerned heavy metals were hyperaccumulated in the plant. The TF elucidates the capability of the plant to translocate the accumulated metals to its other parts. The roots of *E. crassipes* showed the highest translocation capacity for Ni (1.57) as well as Zn (1.30) to other parts. If the value of TF exceeds 1, then it represents the high accumulation efficiency^37,38^, therefore, plants will be considered as the hyperaccumulators for the Ni and Zn. Although the Cd was the highest accumulated metal in the plant, it could have been because of its may be because of its low TF. Whereas, TF values lower than 1 for Cr, Mn, Fe, Cu, and Cd pointed out that this plant's roots act as a non-hyperaccumulator for these heavy metals. Furthermore, the highest MF values were depicted for Mn in both cases which reflects that *E. crassipes* can suitably be used for phytoextraction of Mn as well as for Cd, Zn, Fe, Ni, and Cu. The BAF, TF, and MF of Cr are low in the present study, which implies that roots are limiting the Cr. Moreover, if the BAF ≤ 1.00 then it shows the capability of absorption only rather than accumulation^36,37^. In addition, if the values of BAF, TF, and MF exceed 1, plants can also work for phytoextraction. Furthermore, if the BAF > 1 and TF < 1, represents that plant is a good phytostabilizer as well^35,37,39^. In the present study, it was observed that *E. crassipes* can also work as a good phytostabilizer for Cr, Mn, Fe, Cu, and Cd.

### Human health risk assessment

Freshwater ecosystems are polluted everywhere by anthropogenic activities so it become a prime concern worldwide mainly due to the issues of water quality and seafood contamination. Hence to evaluate the possible health hazards, a health risk assessment was carried out in the form of target hazard quotient (THQ), hazard index (HI), and target cancer risk (TR) by consumption of *Bagarius sp.* from the Narora channel (Table 7). Non-cancer risk is represented by THQ and Cd shows the highest THQ in both adult males (3.21 × 10^−2^) and females (3.66 × 10^−2^) and minimum by Fe in both males (2.03 × 10^−5^) and females (2.31 × 10^−5^) adult individuals respectively. Moreover, the THQ value above 1 indicates that the exposed population could suffer from non-carcinogenic risks in their life duration. In the present study, the THQ for all the concerned metals was below 1, so the *Bagarius sp.* could not pose any non-cancer risk but it shows the level of concern for Cd. Furthermore, the HI is the total THQ, and in the present study, it indicates lower non-cancer risks for males (39.80 × 10^−3^) whereas females (45.38 × 10^−3^) were facing comparatively higher non-cancer risks. This different risk pattern could be due to their low weight because other parameters were the same. In the present study, cancer risk was calculated for Cr and Ni only. For Cd, the carcinogenic slope factor is not available. Ni posed a higher cancer risk to the exposed population than Cr. In males, the TR Ni value was 3.96 × 10^−5^ and in females, it was 4.52 × 10^−5^, while Cr represented 8.54 × 10^−6^ in males and 9.74 × 10^−6^ in females. Between both, groups females were at higher risk for cancer as well. In line with the present investigation, gender differences were also noted by Tchounwou et al.^40^ and Balali-Mood et al.^41^. In general, the toxicity caused by heavy metals leads to several disorders which may be acute as well as chronic. The disorders may be of an immune and nervous system, gastrointestinal, renal disturbances, lesions in vessels and skin, birth defects, and may even lead to cancer. Several authors have reported that simultaneous exposure to a variety of metals either through water or food has synergistic effects^42–44^. Moreover, there are reports on hormonal imbalance caused by Cr and Cd, that both of them interfere with thyroid and steroid metabolism and caused thyrotoxicosis^45^.

**Table 7:** Health risk assessment parameters THQ, HI, and TR.

Heavy metals	THQ		HI		TR	
	Adult male	Adult female	Adult male	Adult female	Adult male	Adult female
Cr	5.69 × 10^−3^	6.49 × 10^−3^	12.51 × 10^−4^	14.32 × 10^−4^	8.54 × 10^−6^	9.74 × 10^−6^
Mn	3.15 × 10^−5^	3.59 × 10^−5^				
Fe	2.03 × 10^−5^	2.31 × 10^−5^				
Ni	1.16 × 10^−3^	1.32 × 10^−3^			3.96 × 10^−5^	4.52 × 10^−5^
Cu	7.17 × 10^−4^	8.17 × 10^−4^				
Zn	8.74 × 10^−5^	9.97 × 10^−5^				
Cd	3.21 × 10^−2^	3.66 × 10^−2^				

### Strategies to minimize heavy metal pollution in the Narora channel

The growing pollution load (heavy metals) of the river Ganga has attracted the attention of researchers as well as others who are concerned with the vulnerability of the environment.

Narora is a town and it is situated on the bank of the river Ganga. According to the Town and Country Planning Department, Uttar Pradesh as per the 2001 census the population of Narora was 20,376 (https://uptownplanning.gov.in/article/en/introduction-of-regulated-area-narora). It has occupied by petrol pumps, drug stores, small-scale sugarcane mills, water pumping and treatment plant, mechanical workshops, intensive agricultural and cropping areas around the bank of canal, etc. Moreover, an Atomic power plant is also present adjacent to this canal. The government made this canal mainly for irrigation of the crop fields of Narora and also to feed the atomic power plant. Additionally, as per the reports of the National Ganga River Basin Authority (NGRBA) Narora town has no sewage facility, consequently leading to the direct release of the town’s wastewater except the power plant into the canal. This further adds to the pollution load of the river Ganga and its canal. Table 8 shows the presence of different heavy metals in the different stretches of the river Ganga.

**Table 8:** Concentrations of different heavy metals in the different stretches and tributary of river Ganga.

Study site	Heavy metal concentrations (μg/L)							References
	Cr	Mn	Fe	Ni	Cu	Zn	Cd	
Narora channel*	0.454	0.156	1.44	1.702	0.265	0.778	0.922	Present study
Allahabad	18	–	–	–	30	122	10	85
Rishiikesh-Allahabad	–	–	–	–	3600	106,300	13,100	103
Rishikesh	–	–	–	36.7	58.1	1349.7	–	114
Berhampore	18	712	1744	84	7	95	2	84
Kanpur	390.8	272.6	27.95	63.7	52.1	49.49	–	69
Haridwar	196	16	–	–	178	219	–	96
Bhagalpur	1090	–	–	120	120	870	ND	98
Diamond Harbour	–	350	560	–	90	710	–	80
Dakshineshwar	22	436	1413	44	8	83	3	84
Ganga Sagar	–	290	320	–	90	520	–	80
Kolkata	–	490	420	–	49	280	–	80
Kaushambi	–	–	600	–	1000	980	–	112
Palta	21	417	2345	53	7	111	3	84
Mirzapur	–	34.25	72.77	67.25	38	94.25	13.37	68
Varanasi	1090	–	150	900	2000	600	160	77
Rishra- Konnagar	0.391	–	–	–	0.322	0.691	0.088	83
Uluberia	24	172	1584	83	6	84	3	84

*In the present study the values are in mg/L.

*ND* not detected.

The present study has already reported the poor water quality condition and poor health status of the indicator organisms of the canal. Although, no non-cancer risk was found but the exposed population may have cancer risk due to Ni and Cr. Furthermore, it too brings about an unhygienic, unhealthy, situation in the town which is threatening public health. Thus, for the abatement of the pollution of this canal or the Ganga river and also to provide healthy conditions there must be a provision of a well-planned sewage/ drainage system in the town.

Besides, it has already been reported that industrial and domestic wastewaters are the predominant sources of heavy metals in the environment^46,47^. In 1986 the government of India launched the Ganga Action Plan intending to clean Ganga and its tributaries unfortunately, they have had little success in achieving their objectives and goals.

Therefore, another way of improving the water quality of this study canal is through phytoremediation. The present study already reported *E. crassipes* as a suitable hyperaccumulator. No doubt, it is a prolific grower and can cause harm to the water body by creating dense mats on the surface, clogging, and blocking it, affecting navigation through the water body, irrigation of the crops, etc. But we have to exploit its hyperaccumulation and phytoextraction capability and its rapid growth can be controlled by time to time mechanical harvesting method and then it can present an attractive source of green, low-cost, remediation tool. In an interesting study by Jones et al.^48^ where they grow the *E. crassipes* plant to explore its phytoremediation potential for heavy metals for the clean-up of the highly polluted tributary of Tawe river, a Nant-Y Fendrod. They conducted experiments in three levels (i) in situ study where water hyacinth was cultured within the river Nant-Y-Fendrod, (ii) bench scale trial where the plant was grown in the polluted river water and in synthetic solutions (iii) bankside study where the plants were grown in the treated river water. Their results were fascinating they successfully removed 21 heavy metals from the water. Among the methods used the bench scale demonstrated promising results with a higher removal rate of Al (63%), Zn (62%), Cd (47%), As (23%), and Mn (22%) whereas in insitu trial the average removal rate for Cd (15%), Zn (11%), and Mn (6%). Another study by Lissy and Madhu^47^ also observed that if it grows collectively in a tank then it showed a 65% removal of heavy metals than in jars. Therefore, the present study suggested that the phytoremediation method by use of *E. crassipes* can be adopted for the abatement of the pollution load of the Ganga river in general and the Ganga canal in particular provided the harvesting of the plant should be done regularly. Moreover, the phytoremediation technique is sustainable, eco-friendly, cost-effective, and as well as requires low maintenance.

The present research investigated high concentrations of heavy metals in Narora channel water. Among heavy metals, Cr, Fe, Ni, and Cd were above the permissible limits. Ni and Cd are represented to be the main culprits which degraded the water quality. All the concerned heavy metals showed significant bioaccumulation in the fish *Bagarius sp.* and aquatic plant *E. crassipes* leading to metal mediated stress and consequent depletion of energy reserves. None of these metals do not pose any non-cancer risk but Cr and Cd raised the concern. Cr and Ni posed low cancer risk to the exposed population. Additionally, gender-specific differences were found in the health risk assessment study. This study clearly shows that water quality surveillance was not carried out in rural areas, as indicated by WQI analysis value. Moreover, techniques like bioremediation, phytoremediation, etc. should be employed time to time to maintain the quality of water and life.

## Materials and methods

### Ethical statement for animal and plant experimentation

All the experiments were permitted by Ministry of Environment and Forests, Government of India under registration no. 714/02/a/CPCSEA which was issued and approved by the institutional ethical committee of Department of Biochemistry, Aligarh Muslim University, Aligarh, India. Moreover, for the collection of samples of fish and plant permissions were obtained and all the procedures were performed in accordance to the guidelines.

### Sample collection

**Figure 4:**
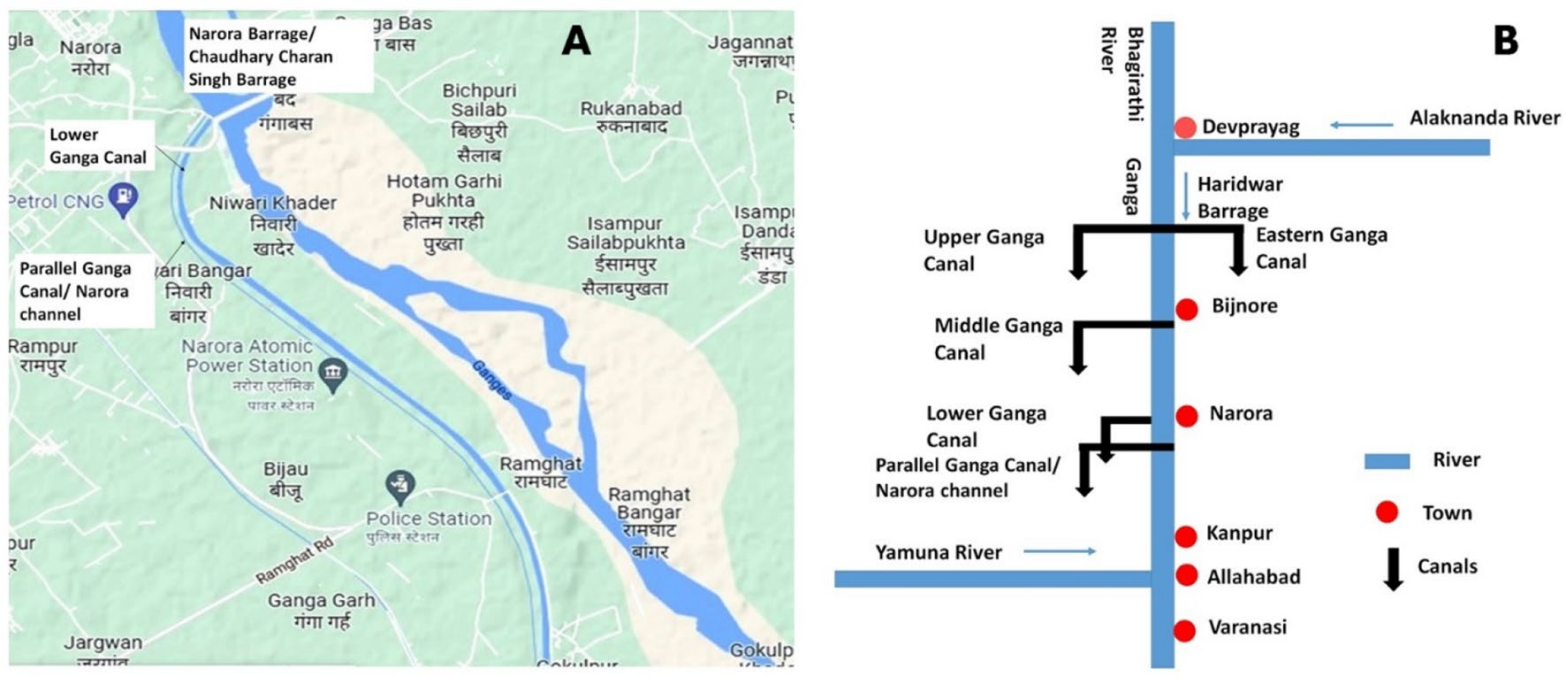
(**A**) Map of study site showing Narora channel; (**B**) Schematic representation of canal system of river Ganga.

Water samples (n = 3), fish *B. bagarius* (n = 15), and plant *E. crassipes* (n = 8) were collected from three different locations that is at the starting, around the midpoint, and at the exit of the Narora channel at Narora, Uttar Pradesh. Being present only at a few spots *E. crassipes* plant was scarce. The reference fish was also collected (n = 15) from another freshwater channel. The Fig. 4A and B shows the study site and schematic representation of different canals of river Ganga including the concerned site respectively.

Different organs of exposed fish like muscle, liver, kidney, and gills were dissected out. Likewise, the plant parts were also separated. All the tissues of the fish and the plant parts were separately oven dried and then powdered in a pestle and mortar. Around 2 g of each sample was taken in a flask and acid digested (HClO_4_ and HNO_3_ in a ratio of 1:4 v/v) at ± 60 °C for estimation of heavy metals by atomic absorption spectrophotometer^35^. The instrument was calibrated using the standard reference dog fish muscle (DORM-2). Moreover, the MPI was computed for fish samples according to the formula of Javed and Usmani^49^ as follows:

$${\text{MPI}}\, = \,\left( {{\text{Cf}}_{{1}} \, \times \,{\text{Cf}}_{{2}} \, \times \,\_\,\_\,\_\,\_\,\_\,{\text{Cf}}_{{\text{n}}} } \right)^{{\text{1/n}}} ,$$

where Cf_1_, Cf_2_ ….. up to Cf_n_ is the amount of the heavy metal ‘n’ in the fish tissue.

The temperature, pH, D.O., total dissolved solids (TDS), turbidity, and alkalinity (CaCO_3_) of test water were assessed according to the APHA^50^ guidelines.

### Analytical method validation

The precision study was described for five replicate of 100 µgL^−1^ concentrations of each metal ion namely Cr, Mn, Fe, Ni, Cu, Zn, and Cd. Moreover, accuracy was established by percent recovery experiments, by spiking the known amount of 5 µg and 10 µg of metal ions (Cr, Mn, Fe, Ni, Cu, Zn, and Cd) in the test water samples.

### Water quality index (WQI)

WQI was calculated with the help of the Indian standards for drinking water^18^ as follows:

1. Weight (wi) was allotted between 1 and 5 to every studied water characteristic (temperature, pH, D.O., TDS, alkalinity, turbidity, Cr, Fe, Mn, Ni, Cu, Zn, and Cd) on the basis of their relative significance on water quality for drinking (Table 2). The highest weightage (5) was given to Cr and Cd due to their remarkable impact on the quality of water, while the least weightage (1) was given to Zn because of its little importance^2^.
2. Relative weight (Wi) calculation was done using the following equation:
  $$Wi=\frac{wi}{\sum_{i=1}^{n}wi}$$
  where, Wi = relative weight of ith parameter, wi = allotted weight of each parameter, n = total number of investigated parameters of water quality.
3. Quality rating scale (qi) calculation was done as per the formula^18^:
  $${\text{qi}}\, = \,{\text{Ci}}\,/\,{\text{Si}}\, \times \,{1}00,$$
  where, qi = quality rating, Ci = concentration of every investigated parameter of water in the test water, Si = Indian permissible limits^18^ established for drinking water.

For determination of WQI, the sub index (SI) was evaluated for every concerned water quality parameter, as follows:

$${\text{SIi}} = {\text{ Wi}} \times {\text{qi}},$$

$${\text{WQI}} = \Sigma {\text{SI}}_{{\text{i - n}}} ,$$

where, SIi = sub-index for ith parameter; Wi = relative weight of ith parameter; qi = rating of the ith parameter, n is the total number of investigated water quality parameters.

### Condition indices

These indices represent the wellbeing of the fish. It includes condition factor (K), hepato-somatic index (HSI), and kidney somatic index (KSI). They were calculated as per the methods of Desai^51^ and Fulton^52^.

$${\text{K}}\, = \,{\text{weight}}\,{\text{of}}\,{\text{fish}}\,\left( {\text{g}} \right)\,/\,{\text{length}}^{{3}} \,\left( {{\text{cm}}} \right)\,{\text{of}}\,{\text{fish}}\, \times \,{1}00$$

$${\text{HSI}}\, = \,{\text{weight}}\,{\text{of}}\,{\text{liver}}\,\left( {\text{g}} \right)\,/\,{\text{fish}}\,{\text{weight}}\,\left( {\text{g}} \right)\, \times \,{1}00$$

$${\text{KSI}}\, = \,{\text{weight}}\,{\text{of}}\,{\text{kidney}}\,\left( {\text{g}} \right)\,/\,{\text{fish}}\,{\text{weight}}\,\left( {\text{g}} \right)\, \times \,{1}00.$$

### Glucose, glycogen, and protein assay

For the collection of serum, blood was centrifuged for 10 min at 3500xg. The glucose amount was determined by using a commercial kit Eco-Pak glucose (Accurex Biomedical Pvt. Ltd., India), and was read at 505 nm on a UV–Vis spectrophotometer (Systronics, 118). Glycogen concentration was estimated via the Anthrone reagent procedure^53^. The total protein amount was determined by Bradford’s^54^ method.

### Assessment of bioaccumulation factor, translocation factor, and mobility factor in plant

The indices BAF, TF, and MF were determined through the following Equations^2^:

$${\text{BAF}}\, = \,{\text{average}}\,{\text{metal concentration }}\left( {{\text{mg}}/{\text{kg}}} \right){\text{ in shoot }}\left( {{\text{root}}\, + \,{\text{stem}}\, + \,{\text{leaves}}} \right)\,/\,{\text{metal concentration }}\left( {{\text{mg}}/{\text{kg}}} \right){\text{ in water}}$$

$${\text{TF}}\, = \,{\text{average metal concentration }}\left( {{\text{mg}}/{\text{kg}}} \right){\text{ in shoot }}\left( {{\text{root}}\, + \,{\text{stem}}\, + \,{\text{leaves}}} \right) \, /{\text{ metal concentration }}\left( {{\text{mg}}/{\text{kg}}} \right){\text{ in root}}$$

$${\text{MF}}\, = \,{\text{average concentration }}\left( {{\text{mg}}/{\text{kg}}} \right){\text{ in receiving level }}/{\text{ metal concentration }}\left( {{\text{mg}}/{\text{kg}}} \right){\text{ in source level}}.$$

### Risk assessment parameters

#### THQ

It represents non-cancerous risk and is dimensionless. It was evaluated using the USEPA region III risk-based concentration table^55^:

$${\text{THQ}}\, = \,\frac{{{\text{Mc}} \times {\text{IR}} \times {1}0^{{ - {3}}} \times {\text{EF}} \times {\text{ED}}}}{{{\text{RfD}} \times {\text{Bw}} \times {\text{ATn}}}}.$$

#### HI

HI is the total of all THQs (USEPA, 2011)

$${\text{HI}}\, = \,{\text{THQCr}}\, + \,{\text{THQMn}}\, + \,{\text{THQFe}}\, + \,{\text{THQNi}}\, + \,{\text{THQCu}}\, + \,{\text{THQZn}}\, + \,{\text{THQCd}}.$$

#### TR

It depicts the cancerous risk and also a dimensionless quantity and was assessed using USEPA region III risk-based concentration table^55^.

$${\text{TR}}\, = \,\frac{{{\text{Mc}}\, \times \,{\text{IR}}\, \times \,{1}0^{{ - {3}}} \, \times \,{\text{CPSo}} \times \,{\text{EF}}\, \times \,{\text{ED}}}}{{{\text{Bw}}\, \times \,{\text{ATc}}}},$$

where, Mc = metal quantity in fish fillet (mg/kg dry weight), IR = ingestion rate (19.5 × 10^−3^ kg/day) for both adult human male and female person, Bw = average body weight taken as 57 kg for adult male and 50 kg for female person Shukla et al.^56^, EF = exposure frequency taken as 365 days/year, ED = exposure duration, 67 years (Expectancy of life of Indian man and woman is about 65 and 68 years respectively). However, for calculation their average was used. (https://countryeconomy.com/demography/life-expectancy/India), ATn = average time for non-carcinogenic exposure is 365 days/year × ED^55,57^, ATc = average time for carcinogenic exposure is 365 days/year × ED^55,57^, RfD = metal reference dose which are as follows Cr = 0.003 mg/kg/day, Mn = 0.14 mg/kg/day, Fe = 0.17 mg/kg/day, Ni = 0.02 mg/kg/day, Cu = 0.04 mg/kg/day, Zn = 0.3 mg/kg/day, and Cd = 0.001 mg/kg/day^57^, CPSo = carcinogenic potency slope for oral dose which is 0.5 mg/kg bw-day^−1^ for Cr and 1.7 mg/kg bw-day^−1^ for Ni^57^.

Among the studied heavy metals only Cr and Ni only were considered for TR calculation as they are carcinogenic. While TR for Cd was not available.

Before calculation of THQ and TR, there are a few things which are supposed to be mentioned below:

- (a) Both ingested dose and absorbed dose of pollutant are equal^58^.
- (b) Pollutants have no effect on cooking^59^.

### Statistical analysis

The study of water quality parameters was done in replicates of three, and that of fish and plant analysis were done in triplicates. The results are presented as mean (mean ± SEM in Tables 2, 3 and 5). Duncan’s multiple range test and Student's t test were used for statistical analysis using SPSS software (version 18). Significance was tested at p < 0.05.

## Data Availability

Data will be available upon the request to the corresponding author.
